# Active surveillance should not be routinely considered in ISUP grade group 2 prostate cancer

**DOI:** 10.1186/s12894-023-01315-5

**Published:** 2023-09-30

**Authors:** Giorgio Gandaglia, Riccardo Leni, Sophie Plagakis, Armando Stabile, Francesco Montorsi, Alberto Briganti

**Affiliations:** 1 Unit of Urology/Division of Oncology; URI, IRCCS Ospedale San Raffaele, Milan, Italy; 2 Vita-Salute San Raffaele University, Milan, Italy; 3 Flinders Medical Center, Adelaide, Australia

**Keywords:** Prostate cancer, Active surveillance, Intermediate risk, Radical prostatectomy, Recurrence

## Abstract

Active surveillance has been proposed as a therapeutic option in selected intermediate risk patients with biopsy grade group 2 prostate cancer. However, its oncologic safety in this setting is debated. Therefore, we conducted a non-systematic literature research of contemporary surveillance protocols including patients with grade group 2 disease to collect the most recent evidence in this setting. Although no randomized controlled trial compared curative-intent treatments, namely radical prostatectomy and radiotherapy vs. active surveillance in patients with grade group 2 disease, surgery is associated with a benefit in terms of disease control and survival when compared to expectant management in the intermediate risk setting. Patients with grade group 2 on active surveillance were at higher risk of disease progression and treatment compared to their grade group 1 counterparts. Up to 50% of those patients were eventually treated at 5 years, and the metastases-free survival rate was as low as 85% at 15-years. When considering low- and intermediate risk patients treated with radical prostatectomy, grade group 2 was one of the strongest predictors of grade upgrading and adverse features. Available data is insufficient to support the oncologic safety of active surveillance in all men with grade group 2 prostate cancer. Therefore, those patients should be counselled regarding the oncologic efficacy of upfront active treatment modalities and the lack of robust long-term data supporting the safety of active surveillance in this setting.

## Introduction

Active surveillance (AS) is the standard of care for patients with low-risk localized prostate cancer (PCa) to postpone or avoid treatment-related side effects without losing the window of curability [1]. A close follow-up with longitudinal evaluations of PSA, digital rectal examination (DRE), and biopsy with or without multiparametric MRI (mpMRI) is mandatory to identify disease progression in a timely fashion. Proper patient selection with the adoption of strict criteria represents one of the cornerstones of this approach. Prospective series supported the long-term oncological safety of AS in men with low-risk disease, where the 15-year cancer-specific mortality-free survival rates exceed 94% [2]. Given the impact on quality of life of side effects of curative-intent treatments, namely erectile, voiding, and bowel disfunctions, AS has been proposed as a valid strategy also in selected men with grade group (GG) 2 (Gleason 3 + 4) PCa [1]. However, its role in this setting is still debated [3–5]. Some authors argued that selected patients with low volume GG 2 could be enrolled in AS protocols due to their favourable pathologic features when managed with radical prostatectomy (RP) and their low risk of disease recurrence after surgery [3, 6, 7]. When looking at real-world data, the use of AS in intermediate risk patients ranges between 4.6 and 15% in the USA (National Cancer Database) and in Sweden, respectively, and varies significantly according to age, demographics, and biopsy features [8, 9]. However, only few prospective studies enrolled intermediate risk patients with GG 2 PCa, and robust long-term data on the oncologic safety of AS in this setting are still limited [10–12]. In the face of such a paucity of data, we aimed at critically reviewing the available evidence on AS in patients with GG 2 PCa. A collaborative non-systematic review of the literature was performed through April 2023 using the medical electronic databases PubMed and Scopus to identify English-language original articles reporting the outcomes of AS in patients with intermediate risk features and GG 2 PCa. The identified studies represented the basis for a narrative review of the literature analysing the role of AS in patients with GG 2 disease.

## Curative-intent treatments reduce the risk of progression in intermediate risk patients: randomized controlled trials

Three randomized trials assessed the role of surgery in the management of PCa patients with localized disease [13–15]. Although none of them included patients managed with contemporary AS criteria, their results can inform physicians on the risk of disease progression among men with intermediate risk PCa managed conservatively. The SPCG-4 study randomized patients with early PCa to RP vs. watchful waiting. Surgery was associated with a 12% absolute risk reduction in cancer-specific mortality at a 23-year follow-up [13]. Similarly, long-term results of the PIVOT trial were recently reported, highliting an effect of surgery on all-cause mortality that was greater among men with intermediate risk disease, with a 13% reduction in the overall mortality rate [14]. The control arm of both these trials was based on watchful waiting with the delivery of palliative therapies after the onset of symptoms, an approach much different from contemporary AS strategies. Therefore, although these studies support the oncologic benefits of surgery in the intermediate risk setting, their results cannot be generalized to contemporary men managed with AS. Moreover, both trials did not specifically report the outcomes of individuals with GG 2 disease, where intermediate risk patients might have a heterogeneous prognosis [3]. Finally, these studies enrolled patients diagnosed in the pre-PSA era who might have a higher disease burden compared to contemporary patients.

The ProtecT study randomized individuals with screening-detected clinically localized PCa to active monitoring vs. RP vs. radiotherapy, approximately 25% of participants had features of intermediate risk disease according to contemporary risk-stratification tools [15]. This study failed to show a benefit in terms of 15-year PCa-specific survival associated with RP or radiotherapy as compared to active monitoring. Nonetheless, the rates of disease progression and metastases were higher among men managed with active monitoring and this was mainly driven by intermediate- and high-risk patients. This could theoretically translate into a survival benefit associated with curative-intent treatments in these groups at longer-term follow-up. Of note, of the 133 men allocated to active monitoring, who were alive and not treated by the end of follow-up, only 13% had intermediate risk features, and only 10% had GG 2 PCa at diagnosis. One of the arguments of the supporters of AS in the intermediate risk setting is that active monitoring in the ProtecT trial did not include follow-up prostate biopsies. Therefore, one might hypothesize that the adoption of more stringent AS protocols that include confirmatory and follow-up biopsies might have allowed for the early identification and cure of men who eventually developed metastases. However, the active monitoring protocol of the ProtecT study was based on serial PSA measurements which theoretically allowed for the delivery of curative-intent therapies at the time of disease progression, where more than 50% of the individuals included in this group eventually received surgery or radiotherapy.

## The presence of grade group 2 disease is associated with an increased risk of disease progression and metastases in AS cohorts

A summary of relevant studies reporting the oncologic outcomes of ISUP biopsy GG 2 PCa patients managed with AS is depicted in Table 1. Of note, only four historic prospective academic cohorts included intermediate risk patients who were mainly affected by GG 2 disease: the Sunnybrook Health Sciences Centre (Toronto, Canada), the Royal Marsden Hospital (London, UK), the University of California San Francisco (UCSF, San Francisco, USA), and the multicentre Canary PASS cohort (10 centres in the USA and Canada).

**Table 1:** Oncologic outcomes of grade group 2 Prostate Cancer (PCa) patients enrolled in active surveillance (AS) protocols

Cohort	Type	Years	Total nr.	IR (GG2)	Median follow-up, yrs (IQR)	TFS	MFS	CSS
Toronto Sunnybrook (Musunuru et al., 2016, Yamamoto et al., 2016)	Prospective	1995–2013	945	213 (102)	7 (4–10)	15y, IR vs. LR: 47.8% vs. 58.2%	15y, IR vs. LR: 82.2% vs. 94.6%	15y, IR vs. LR: 88.5% vs. 96.7%
Royal Marsden (Selvadurai et al., 2013)	Prospective	2002–2011	471	88 (33)	6 (NR)	5y: 70% (entire cohort)	Metastases reported in two patients, one of which had Gleason 3 + 4 at diagnosis	5y: 96% (entire cohort)
UCSF (Maggi et al., 2020)	Prospective	1990–2018	1450	216 (147)	6 (4–10)	7y, GG2: 46%	7y, GG2: 96%	7y: >99% (entire cohort)
Multicenter Canary PASS (Weismann Malaret et al., 2022)	Prospective	2008–2020	1728	NR (154)	6 (3–9)	5y, GG2 vs. GG1: 42% vs. 66%	Six pts with GG1 developed metastases, none with GG2.	NR
MSKCC (Carlsson et al., 2020)	Retrospective	2000–2017	219	219 (219)	3 (2–5)	5y: 61%	5y: 100%	5y: 100%
UCLH (Stavrinides et al., 2020)	Retrospective	2004–2017	672	NR (148)	5 (3–7)	5y: MRI visible GG2 34%; non-visible GG2 63%	Lower in MRI visible GG2 (p = 0.01)	Lower in MRI visible GG2 (p = 0.001)

**Abbreviations**: IR: intermediate risk; LR: low risk; GG2: grade group 2; TFS: treatment free survival; MFS: metastases free survival; CSS: cancer specific survival; NR: not reported; UCSF: University of California San Francisco; PASS: Prostate Active Surveillance Study; MSKCC: Memorial Sloan Kettering Cancer Center; UCLH: University College London Hospital; MRI: magnetic resonance imaging.

The Sunnybrook cohort included 213 (22.5%) intermediate risk patients, 60% of which had biopsy Gleason 7 PCa (102 had Gleason score 3 + 4) and the remaining had PSA between 10 and 20 ng/ml or Gleason score ≥ 4 + 3. Patients with intermediate risk PCa had 15-year metastases-free survival rates of 84% and lower cancer-specific free survival compared to the low-risk setting (89 vs. 97%, respectively) [12]. Of note, the presence of a Gleason 3 + 4 was associated with a 4-fold higher risk of dying from PCa compared to low-risk PCa [16]. This is in line with what was reported for men managed with AS within the Goteborg screening trial, where intermediate risk patients had a 4.8-fold increased risk of AS failure compared to those with very low-risk disease and lower 15-years metastases-free survival (90% vs. 98%) [17].

The Royal Marsden Hospital protocol included only 33 men (7%) with GG 2 PCa and failed to show an increased risk of being treated with curative-intent treatments at a median follow-up of 5.7 years. However, Gleason 7 was the strongest predictor of adverse pathology at RP [11]. The relatively small number of patients with GG 2 disease included in this cohort limits its generalizability.

Approximately 10% of patients managed with AS within the UCSF had Gleason 3 + 4 disease. In their landmark publication, Cooperberg et al. reported that patients with intermediate risk disease were not at increased risk of progression (54 vs. 61% for low- vs. intermediate risk, respectively) or active treatment (30 vs. 35%) [18]. An update that included 124 men with Gleason 3 + 4 disease demonstrated that a higher grade at biopsy was associated with a 1.4-fold increased likelihood of receiving active therapies at 5-year follow-up. Moreover, individuals with GG 2 PCa had higher BCR rates after RP compared to those with GG 1 disease [19]. The authors retrospectively reviewed the outcomes of GG 1 and 2 patients enrolled in AS at their institution and confirmed that men with GG 2 PCa had lower metastases-free survival rates at a median follow-up of 77 months compared to those with GG 1 disease. At multivariable analyses, a biopsy GG 2 was associated with a risk of metastases which was approximately 20-fold higher compared to patients with GG 1 PCa [20].

In the multicentre Canary PASS cohort, 9% of men included had intermediate risk PCa, and although the five-year reclassification rates were similar between GG 2 and 1 disease (30% vs. 37%), a higher proportion of men with GG 2 were treated at 5 years (58% vs. 34%, p < 0.001). Moreover, among men without grade reclassification, those with initial GG 2 switched to treatment more often than those with GG 1. Both adverse pathology and risk of BCR were similar between men who received delayed RP after initial surveillance [21].

A retrospective study reported the outcomes of more than 200 patients with GG 2 disease managed with AS at the Memorial Sloan Kettering Cancer Center (MSKCC). The authors report a 5-year treatment-free survival rate of 61% [22], which is substantially lower as compared to what reported in the same series when evaluating GG 1 patients (76%) [23]. Interestingly, up to 30% of men with GG 2 PCa at MSKCC underwent curative-intent treatments due to a change in patient preference.

Finally, the University College London Hospital (UCLH) recently reported the outcomes of an AS program where biopsies were omitted in favour of MRI monitoring [24]. In particular, patients received a baseline MRI and were monitored using mpMRI and PSA at 12-months. A subsequent mpMRI was performed at 24 months in men with mpMRI-visible lesions while the use of imaging was based on PSA values in men with non-visible lesions. The retrospective study included 672 patients with 148 (22%) being GG 2. The presence of GG 2 as well as MRI-visible lesions represented independent predictors of AS discontinuation and treatment at 5-year follow-up. Finally, evidence from several population-based analyses supported the notion that men with GG 2 initially managed with AS might have worse oncologic outcomes compared to low-risk patients [25–27]. In a large, population-based analysis from the Surveillance, Epidemiology and End Results (SEER) database, among 166,244 men who received either upfront treatment, AS, or watchful waiting, those with GG 2 managed with AS (approximately 2%) had worse overall survival (88% vs. 94%) compared to men with GG 1 [27].

Baboudjian et al. recently summarized findings of AS in intermediate risk PCa in a systematic review and meta-analysis, demonstrating higher risk of metastases and PCa-related mortality for patients with intermediate risk features [28]. However, when excluding men with GG ≥ 3, they could not demonstrate any difference in terms of deferred treatment, metastases, and mortality. Moreover, as acknowledged by the Authors, no discrimination between GG 1 vs. GG 2 was possible due to the nature of the selected studies.

Taken together, these findings highlight that AS in men with intermediate risk features carries a non-negligible risk of disease progression and curative-intent treatment. Moreover, the 15-year metastases-free survival rate can be as low as 85% in intermediate risk patients managed with AS. Therefore, patients with GG 2 PCa, and those with intermediate risk features, who are interested in expectant management should be counselled regarding the increased risk of disease progression and adverse outcomes after curative-intent therapies.

## Are we able to identify biopsy grade group 2 PCa patients affected by an indolent disease who could be safely considered for AS?

When considering men treated with RP, who were otherwise eligible for AS, adverse pathology should be defined as the presence of non-organ confined disease, or GG ≥ 3, or lymph node invasion [29]. As such, the presence of GG 2 PCa at final pathology in AS candidates who were treated with RP does not represent *per se* an adverse prognostic factor for disease recurrence after treatment. However, studies testing this association in the AS setting are lacking, therefore, these notions cannot be directly applied for patients with GG 2 disease managed on AS.

Several retrospective studies which included patients with low- and intermediate risk disease undergoing RP evaluated predictors of favourable pathology to propose novel AS criteria based on individual characteristics. Up to 25% of patients with low-volume intermediate risk PCa defined as the presence of ≤ 2 cores of GG 2 PCa, PSA < 20 ng/ml harbour adverse pathologic features at RP [30], and the presence of biopsy GG 2 disease is associated with a 3-fold increase in the risk of extraprostatic extension compared to lower grade disease [31]. Similarly, a large study evaluating more than 5,200 low- and intermediate risk patients managed with RP demonstrated that GG 2 doubled the risk of harbouring adverse pathology [32]. Of note, the presence of GG 2, as well as other favourable disease features (namely, PSA < 10 ng/ml, ≤ 2 positive cores, non-palpable disease) conferred a risk higher than 18% of adverse pathology according to a novel risk score to identify AS candidates. The 18% cut-off was proposed to increase the proportion of potentially eligible patients without compromising oncologic control and, therefore, the presence of GG 2 disease alone potentially excluded patients from being considered for AS. Recently, efforts in estimating the added prognostic value of length of Gleason pattern 4, rather than its ratio compared to pattern 3 have been undertaken [33, 34]. Albeit a signal towards an increased clinical utility of measuring total length of pattern 4 was observed, no significant differences in terms of net benefits were demonstrated. Perera et al., however, concluded that in terms of risk-stratification for patients with GG 2 disease on AS, length of pattern 4, rather than its proportion, may be of added clinical utility to establish cut-offs for inclusion of those patients [34].

The advent of novel imaging modalities such as mpMRI for diagnosis and staging might alter this scenario. Lantz et al. recently reported that, although GG 2 PCa is still associated with misclassification at RP, a higher nomogram-calculated threshold can be used to select AS candidates among men diagnosed with mpMRI [35]. Moreover, the use of MRI-targeted biopsy can improve patient selection, where a recent study demonstrated that men with GG 2 disease diagnosed with MRI-targeted plus systematic biopsy had substantially lower adverse pathology rates compared to their counterparts diagnosed with systematic biopsy alone [36]. Therefore, mpMRI represents a promising tool for the selection of GG 2 PCa patients who might be considered for AS. However, prospective studies supporting the safety of this approach in patients managed with AS are still missing.

## Summary

The use of AS has been proposed in selected intermediate risk PCa patients with GG 2 disease to reduce the risk of treatment-related side effects without compromising oncologic outcomes [4, 6]. Over the last few years, the adoption of this approach roughly tripled from 1.6% to 2010 to 4.6% in 2016 in the USA [9]. Recently, an updated report of the ProtecT trial questioned the benefits of aggressively treating screening-detected, clinically localized PCa compared to an expectant strategy. Such position was supported by the evidence of very low incidence of PCa specific mortality regardless of the allocation arm, reflecting the long natural history of this disease [15]. However, the evidence supporting the oncologic safety of AS in GG 2 PCa is still limited, especially in the era of MRI and novel imaging techniques. Indeed, available prospective cohorts include mainly highly selected patients with low-risk disease, where intermediate risk patients represent only a minority of the individuals. Overall, the long-term results of these studies demonstrate that patients with GG 2 disease are at increased risk of disease progression, of receiving curative-intent therapies and of experiencing metastases compared to those with GG 1 PCa [3, 6, 18, 21]. In particular, up to 50% of GG 2 patients were eventually treated 5 years after AS enrolment and the metastases-free survival rate for was as low as 85% at 15-year follow-up. Therefore, these studies demand caution when considering men with GG 2 PCa for inclusion in AS protocols due to the higher risk of progression and metastases at long-term follow-up.

Besides prospective AS cohorts, other authors reported that a GG 2 disease roughly doubles the risk of adverse pathology if patients with low- or intermediate risk PCa were treated with RP [30–32]. When considering patients diagnosed with systematic biopsy, the presence of GG 2 was found associated to grade upgrading in more than 25% of patients, regardless of other favourable disease characteristics, a percentage that warrants caution when considering those men for AS [32]. However, the use of mpMRI and MRI-targeted biopsy might mitigate this risk allowing for proper patient selection and reducing the rate of adverse pathology in patients with GG 2 PCa managed with RP [35, 36]. Nonetheless, the long-term results of prospective studies including GG 2 patients selected with mpMRI/MRI-targeted biopsy and managed with AS are still lacking. Therefore, the generalizability of indirect inferences on the safety of AS in men with GG 2 PCa diagnosed at mpMRI and targeted biopsy is not warranted in contemporary patients. Highly selected patients with intermediate risk disease who are interested in AS should be counselled regarding the efficacy of curative-intent treatments such as RP [13, 14, 37]. Moreover, physicians should clearly detail the lack of robust data and long-term follow-up on the safety of AS in the management of PCa in this setting, as well the increased risk of metastases in case of initial expectant management.

Nevertheless, physicians shall also carefully weight benefits and harms of aggressive treatment modalities, considering age and comorbidities in adjunction to clinical and pathologic features of intermediate risk PCa [38]. Some may argue that an old man with serious comorbidities and a minimal component of pattern 4 may avoid any form of treatment, we strongly agree on that, however he would be an ideal candidate for watchful waiting rather than AS.

In the next future we can expect that the widespread availability of mpMRI and molecular imaging techniques such as Prostate Specific Membrane Antigen Positron Emission Tomography (PSMA PET), with consequent improved ability to correctly stratify patients will allow for a better classification of patients with GG 2 disease [39]. PSMA PET may have a pivotal role in the early detection of patients with intermediate risk disease who may benefit from upfront curative treatments rather than AS [40]. Moreover, novel metrics to quantify the amount of pattern 4 in the biopsy will be of added value in the clinical decision making for the selection of patients with GG 2 for AS [34]. Until then, times are not yet ready to see a widespread adoption of management with AS for all patients with GG 2 PCa [41].

## Conclusions

AS is the preferred management for patients with low-risk localized PCa and some authors advocated the use of this approach in men with favourable GG 2 disease. However, available data is insufficient to support the oncologic safety of AS in this setting. At the time being, patients with GG 2 PCa at prostate biopsy should be counselled regarding the oncologic efficacy of upfront treatment and the lack of robust long-term data supporting the safety of AS in this setting. Most importantly, these men should be counselled on the almost 3-fold increase in the risk of developing metastases if managed expectantly. Until optimal risk stratification tools are available, such as novel imaging techniques and contemporary metrics to quantify the amount of pattern 4 in biopsies, AS shall not be routinely implemented in all patients with GG 2 disease.

## Data Availability

Not applicable.

## List of abbreviations

AS: Active surveillance
DRE: Digital Rectal Examination
GG: Grade Group
mpMRI: Multiparametric Magnetic Resonance Imaging
PCa: Prostate Cancer
PSA: Prostate Specific Antigen
PSMA PET: Prostate Specific Membrane Antigen Positron Emission Tomography
RP: Radical Prostatectomy
SEER: Surveillance, Epidemiology and End Results
